# Evaluation of [^18^F]MNI-1054, a novel PET ligand for lysine-specific histone demethylase 1A (LSD1), in non-human primates

**DOI:** 10.1186/s13550-025-01350-3

**Published:** 2025-12-06

**Authors:** Yoann Petibon, Akihiro Takano, Adam J. Schwarz, Ozlem Yardibi, Christine Sandiego, Olivier Barret, Cristian Constantinescu, Johannes Tauscher, Paul McQuade

**Affiliations:** 1 Takeda Development Center Americas, Inc., 125 Binney St, MA 02139 Cambridge, USA; 2 Takeda Pharmaceutical Company Ltd., Osaka, Japan; 3 Invicro LLC, New Haven, CT USA

**Keywords:** PET, LSD1, Occupancy, Non-human primate

## Abstract

**Background:**

The aim of this study was to characterize the novel LSD1-specific PET radiotracer [^18^F]MNI-1054 in rhesus monkeys and to utilize it to evaluate occupancy of TAK-418, a novel LSD1 inhibitor. To accomplish this, eleven 180-minute dynamic brain PET scans were performed in two rhesus monkeys (1 male/1 female), including baseline scans and a self-block with unlabeled MNI-1054 (3 mg/kg) to assess total levels of specific binding. Displacement and blocking studies with TAK-418 were performed to confirm irreversible binding and to evaluate the dose-occupancy relationship of TAK-418. Scans were also acquired 24- and 48-hours post-TAK-418 dosing to assess LSD1 repopulation rates. Additional baseline and blocking studies with 3.0 mg/kg TAK-418 were acquired in a male monkey to evaluate peripheral binding and occupancy in the testes, an organ with high LSD1 expression. Lastly, whole-body scans were obtained from two animals (1 male/1 female) to evaluate dosimetry.

**Results:**

Across studies, [^18^F]MNI-1054 fraction in plasma was ~42% at 30 min and ~14% at 180 min after injection. Tracer kinetics were accurately modeled using an irreversible two-tissue compartment model, yielding *K*_*i*_ as the binding endpoint. The highest specific signal was found in the cerebellum, and the neuroanatomical signal profile was consistent with that of LSD1 expression. The specific signal was blocked in a dose-dependent fashion by the molecularly distinct LSD1 inhibitor TAK-418, with *O*_max_ = 95.6% and *ED*_50_ = 0.0224 mg/kg in cerebellum. Scans at later time points yielded an LSD1 repopulation half-life estimate of 12.28 h. Evidence of significant LSD1 expression and occupancy was found in testes with 3.0 mg/kg TAK-418, however point occupancy levels could not be reliably estimated from *K*_*i*_. The estimated whole-body effective dose was ~0.027 mSv/MBq, with the gallbladder wall being the limiting organ (0.18 mSv/MBq).

**Conclusions:**

[^18^F]MNI-1054 displayed acceptable brain penetrance, kinetics and LSD1 specificity as well as an acceptable dosimetry. Overall, these findings show its suitability as a viable PET probe to assess the binding profile of LSD1 inhibitors in the brain and support further evaluation in humans.

**Supplementary Information:**

The online version contains supplementary material available at 10.1186/s13550-025-01350-3.

## Background

Epigenetic dysregulation, which refers to reversible modifications to DNA and associated proteins that alter gene expression without changing the underlying DNA sequence, is recognized as a key contributor to the pathophysiology of neurodevelopmental and neurological disorders. Epigenetic mechanisms mediate the complex interplay between genetic and environmental factors, beginning during critical periods of brain development and continuing throughout the lifespan [1]. This control is often exerted through chromatin remodeling, where chemical modifications to histone proteins alter the accessibility of DNA for transcription. In particular, the methylation of histone H3 lysine 4 (H3K4) is a critical epigenetic mark for learning and memory [2] and its reduction has been implicated in the pathophysiology of several central nervous system (CNS) disorders including autism spectrum disorders, schizophrenia and Huntington’s disease [3–6].

Lysine-specific demethylase 1 (LSD1) is a key enzyme controlling H3K4 methylation, playing a vital role in neuronal transcriptional programs, plasticity, and survival [7]. Preclinical studies have shown that LSD1 inhibitors increase H3K4 methylation levels in the brain and rescue cognitive deficits in mouse models of neurodevelopmental disorders [8–10]. The indispensable nature of LSD1 is underscored by evidence that its inducible deletion in adult mice causes severe hippocampal and cortical degeneration, paralysis, and memory loss [11]. Studies have shown that LSD1 is mislocalized and sequestered by pathological protein aggregates like Tau and TDP-43, potentially compromising its essential function in the nucleus and contributing to neurodegeneration [12]. Overall, given its regulation of critical cellular processes through both demethylase-dependent and independent functions, LSD1 represents a compelling therapeutic target across a broad spectrum of CNS disorders [13].

Successful clinical translation of LSD1-targeted therapeutics, however, depends on confirming that candidate molecules engage the target in the living human brain. By using highly selective brain-penetrant radioligands that bind to the target of interest, positron emission tomography (PET) allows for the non-invasive confirmation that a drug has reached its intended target and modulates it as expected in the brain, providing critical pharmacodynamic data than can inform drug development and confirm a therapy’s mechanism of action. To non-invasively assess target engagement and dose-occupancy relationships of small-molecule LSD1 inhibitors, a rational design effort was undertaken to identify a small-molecule PET radiotracer for LSD1 [14, 15]. In a preliminary PET study [15], the leading candidate molecule -previously referred to as [^18^F]1 g and [^18^F]-T-914- was reported to exhibit specific binding in brain regions with known high LSD1 expression and displayed favorable kinetics for PET quantification.

The aim of the studies reported here was to more thoroughly characterize this radiotracer (renamed [^18^F]MNI-1054) in non-human primates and evaluate its suitability for human translation. We employed a novel LSD1 inhibitor, TAK-418 [10, 16], as a test article that competitively binds to the same site as [^18^F]MNI-1054. Specifically, and noting that both [^18^F]MNI-1054 and TAK-418 bind irreversibly [10, 15], we assessed in non-human primates: (1) the brain distribution and kinetics of [^18^F]MNI-1054, (2) the dose-occupancy relationship of TAK-418 via blocking studies, (3) the LSD1 enzyme repopulation rate via additional scans at later time points, (4) peripheral LSD1 expression and occupancy in the testes and (5) the whole-body distribution and dosimetry profile of [^18^F]MNI-1054.

## Materials & methods

### Animal care and monitoring

Four rhesus monkeys (macaca mulatta; 2 males (M2, M3) and 2 females (M1, M4) were used as research subjects for imaging studies in brain (M1, M2), testes (M3) and dosimetry (M2, M4). The animals were housed and imaged at Yale University PET Center (New Haven, CT). Studies were carried out under institutional animal care protocols complying with Federal regulations. Animal care approval and oversight was provided by Yale University’s Institutional Animal Care and Use Committee.

Animals were fasted for 8–12 h before each imaging session. At 2 h before radiotracer injection, animals were anesthetized with ketamine (7–15 mg/kg) and glycopyrrolate 0.005–0.01 mg/kg IM, transferred to the PET camera, and immediately intubated with an endotracheal tube for continued anesthesia with 1–3.5.5% isoflurane administered through a breathing circuit. An intravenous line was placed for radiotracer injection and test article administration during the blocking studies. An arterial line was placed for blood sampling. Body temperature was kept at 37–38 °C using a heated water blanket. Vital signs, including heart rate, blood pressure, respiration rate, oxygen saturation and body temperature, were monitored at least every 10–15 min during each study.

### Radiotracer synthesis

[^18^F]MNI-1054 was synthesized as previously described [15], with description of procedures provided in Supplement along with reaction scheme (Fig. S.1.).

### PET brain imaging

[^18^F]MNI-1054 brain scans were acquired in M1 and M2 as summarized in Table 1. All brain scans were collected over 3 h on a microPET Focus 220 PET scanner (Siemens), with injected doses in the range 167.61–185 MBq. A transmission scan with an external Germanium-68 source was performed prior to the emission scans to estimate attenuation correction coefficients [17]. Discrete arterial blood samples (*n* = 18–21) were taken throughout the scans to measure the parent fraction, plasma free fraction as well as total plasma and whole-blood activity for tracer kinetic modeling. Detailed procedures are provided in the Supplement.

**Table 1:** Summary of brain PET imaging studies

Animal	Sex	Purpose of scan [cold compound administered]	TAK-418 or MNI-1054 dose (mg/kg)	Timing of PET scan following cold compound dose (h)	[^18^F]MNI-1054 injected dose (MBq)
M1	F	Baseline [-]	-	-	170.94
		Displacement [TAK-418]	3	−1.5	180.56
		Self-block [MNI-1054]	3	0.08	179.82
		Blocking [TAK-418]	3	2	185
		Blocking [TAK-418]	0.3	2	167.61
		Blocking [TAK-418]	0.01	2	184.63
M2	M	Baseline [-]	-	-	178.71
		Blocking [TAK-418]	0.03	2	173.9
		Blocking [TAK-418]	0.005	2	183.52
		Enzyme repopulation [TAK-418]	0.3	24	167.61
		Enzyme repopulation [TAK-418]	0.3	48	183.52

M1 and M2 each completed a scan at baseline to assess brain penetration and kinetics and to serve as reference for the blocking and displacement studies. A self-block study with cold 3 mg/kg MNI-1054, starting 5 min prior to [^18^F]MNI-1054 administration, was conducted in M1 to assess regional differences in non-specific binding.

Blocking studies with the LSD1 inhibitor TAK-418 (0.005–3.0.005.0 mg/kg) were performed to measure the dose-occupancy relationship for this compound. For blocking studies, TAK-418 was administered intravenously 2 h prior to [^18^F]MNI-1054. Prior to conducting these blocking studies, a displacement study with TAK-418 (3 mg/kg) was performed to confirm irreversible tracer binding, in which TAK-418 was administered 1.5 h post [^18^F]MNI-1054 administration. For all studies involving TAK-418, the test article was administered intravenously over 5 min. Due to the irreversible binding of these compounds to LSD1, the time taken for the enzyme to re-populate was investigated by imaging one animal (M2) with [^18^F]MNI-1054 at 24 and 48 h post TAK-418 treatment (0.3 mg/kg). Each animal also underwent a high-resolution T1-weighted structural brain MRI scan with a 3-D MPRAGE sequence on a separate day for anatomical reference.

### Brain PET processing and kinetic analysis

Emission data were reconstructed with filtered back projection into a series of 45 temporal frames (6 × 30, 3 × 60, 2 × 120, 34 × 300 s). For each frame, reconstructed images had dimensions of 256 × 256 × 95, voxel size of 0.949 × 0.949 × 0.796 mm and units of kBq/mL. All standard corrections were applied including normalization, random, scatter, dead-time, and attenuation. Dynamic brain PET images were analyzed using an in-house processing pipeline incorporating functionalities from FSL [18] and AFNI [19] as well as custom developed Matlab scripts (Mathworks Inc., Natick, MA, USA). Key steps of the workflow included rigidly aligning the PET images to the same animal’s brain MRI scan and spatially normalizing the MRI volume to the INIA19 [20] and NMT2.0 [21, 22] rhesus MR templates. Volumes of interest (VOIs) masks defined in rhesus MR template spaces were then inverse warped and applied to the PET images in their native orientation to compute time-activity curves (TACs) for the following regions: occipital lobe, temporal lobe, frontal lobe, parietal lobe, olfactory cortex, amygdala, cingulate, hippocampus, thalamus, hypothalamus, cerebellar grey matter, pons, striatum, nucleus accumbens and cerebral white matter. Lobar VOI masks were restricted to grey matter only. All VOI masks were derived from the INIA19 atlas, with the exception of the pons mask which was obtained from the NMT2.0 atlas (Fig. S.2.). The average activity concentration (kBq/mL) within each VOI was determined and TACs representing the regional brain activity concentration over time were generated. Brain TACs and images were presented in SUV units [g/mL] by normalizing activity concentration data [kBq/mL] by the animal’s weight [kg] and injected activity [MBq].

To generate arterial input functions, [^18^F]MNI­1054 parent fraction measurements in plasma were first fitted to a sum of decaying exponentials using the Levenberg-Marquardt algorithm [23]. The total arterial plasma activity concentration measurements were then multiplied by the resulting continuous parent fractions to yield the final input functions for kinetic modeling.

Regional TACs were modeled with an irreversible 2-tissue compartmental model (‘2TCM’) (*k*_4_ = 0) with the arterial input function to determine the tracer binding parameters *K*_*i*_ [mL/cm^3^/min] (where *K*_*i*_*= K*_*1*_. *k*_3_/(*k*_2_ + *k*_3_)) and *λ.k*_3_ (where *λ = K*_*1*_*/k*_*2*_), a parameter independent of blood flow and proportional to LSD1 binding site density via *k*_3_. When *K*_*i*_ is not flow-limited (i.e., when *k*_2_ > >*k*_3_), *K*_*i*_ likewise becomes proportional to *k*_3_ and independent of flow so that it can be used as the primary outcome measure reflecting the density of LSD1 sites. As an alternative to compartmental modeling, *K*_*i*_ values were estimated with Patlak graphical analysis [24] likewise utilizing arterial input functions. All kinetic analysis methods assumed a contribution of whole-blood radioactivity to the PET signal fixed at 0.05.

The time stability of *K*_*i*_ estimates for the irreversible 2TCM was evaluated by truncating the full 180-min scan in 30-min increments down to 60 min. The bias of *K*_*i*_ estimates was calculated for each scan duration taking the full 180-min scan as reference.

Regional occupancy due to TAK-418 or unlabeled MNI­1054 as measured with [^18^F]MNI­1054 was computed as the percent change relative to the baseline value of *K*_*i*_, i.e. $\:Occ\left(\%\right)=100\times\:\frac{{K}_{i}^{Baseline}-{K}_{i}^{Drug}}{{K}_{i}^{Baseline}}$ where the drug was either TAK­418 or MNI­1054. An *E*_max_ model with maximum occupancy constrained to a value less than 100% was fitted to the dose-occupancy plot to estimate *O*_max_ (maximal occupancy) and *ED*_50_ [mg/kg] representing the injected TAK-418 dose yielding 50% LSD1 occupancy.

### PET imaging of the testes

It has been reported that testes have high levels of LSD1 expression [25]. To investigate whether [^18^F]MNI­1054 would demonstrate specific binding in peripheral tissue similar to the brain, a baseline and blocking study with TAK-418 (3 mg/kg) were performed in M3 with the testes in the field of view using a Biograph mCT camera (Siemens Healthineers). TAK-418 (3 mg/kg) was administered as an intravenous bolus over 5-min, starting 120 min prior to [^18^F]MNI-1054 administration. Arterial blood samples were also extracted, with sampling times comparable to those of the brain studies.

### Whole-body PET imaging

Dynamic whole-body PET was conducted in one male (M3, 15.4 kg) and one female (M4, 6.8 kg) rhesus to study the biodistribution and radiation dosimetry of [^18^F]MNI-1054. Whole-body images were acquired over ~4 h (17–19 passes, 4 bed positions) using a Biograph mCT PET/CT scanner (Siemens). The images in the series were subsequently reconstructed in 400 × 400 × 300 pixel arrays with pixel size: 2 × 2 × 2 mm^3^ using the iterative True-X algorithm (4 iterations, 21 subsets) provided by the camera manufacturer, incorporating point spread function modeling and corrections for random, scatter, normalization, dead-time and attenuation. Prior to PET, each animal received a whole-body CT for correcting attenuation and scatter as well as to assist in anatomical localization of organs of interest.

### Whole-body image analysis and radiation dosimetry

Reconstructed whole-body PET volumes were transferred to PMOD software (PMOD Technologies, Zurich, Switzerland) for analysis. Decay correction was first removed to facilitate the calculation of radioactive disintegrations from dynamic data. Images were reviewed for assessment of body organ radioactivity distribution. VOIs were manually drawn to capture the following organs: brain, heart wall, lungs, gallbladder, liver, kidneys, spleen, testes (male), urinary bladder, back vertebrae, and intestines, and defined dynamically to account for any animal movement or shift in organ position or shape between acquisitions. Total activity (kBq) within each VOI was determined and organ TACs were generated and expressed as percent of the injected activity.

The number of disintegrations per unit activity administered in each source organ (i.e., the “residence times” or “time-integrated activity coefficients”), τ, were computed from the areas under the non-decay corrected TACs via the trapezoidal method. The area under the curve from the end of imaging to infinity was calculated with the assumption of physical decay only following the last imaging time point. Residence times were converted to adult human values using conversion factors based on differences in organ and body weights between both species. The ICRP 100 HAT gastrointestinal (GI) tract model was applied to estimate residence times in the left, right, and sigmoid (i.e., rectum) colon and small intestine, with the assumption that activity entered the GI tract via the small intestine with no reabsorption. The intestinal decay-corrected time-activity curve was used for estimating the fraction of the radioactivity entering the intestine during imaging. Red marrow residence times were calculated based on assumption that vertebrae contain ~40% of total red marrow.

OLINDA 2.0 software (Hermes Medical Solutions) was used to estimate the organ and whole-body radiation absorbed doses. OLINDA 2.0 performs internal dose calculations based on MIRD and Radiation Dose Assessment Resource (RADAR) methodology. ICRP-89 adult male (73 kg) and female (60 kg) models were used to calculate the s-factors. All other MIRD assumptions about the homogeneity of source organ distribution were employed. The effective doses were computed with biological tissue weighting factors defined in the ICRP Publication 103 [26].

## Results

All animals tolerated the scan procedures without adverse effects being observed from the radiotracer or test articles.

### [^18^F]MNI­1054 in arterial blood

**Fig. 1:**
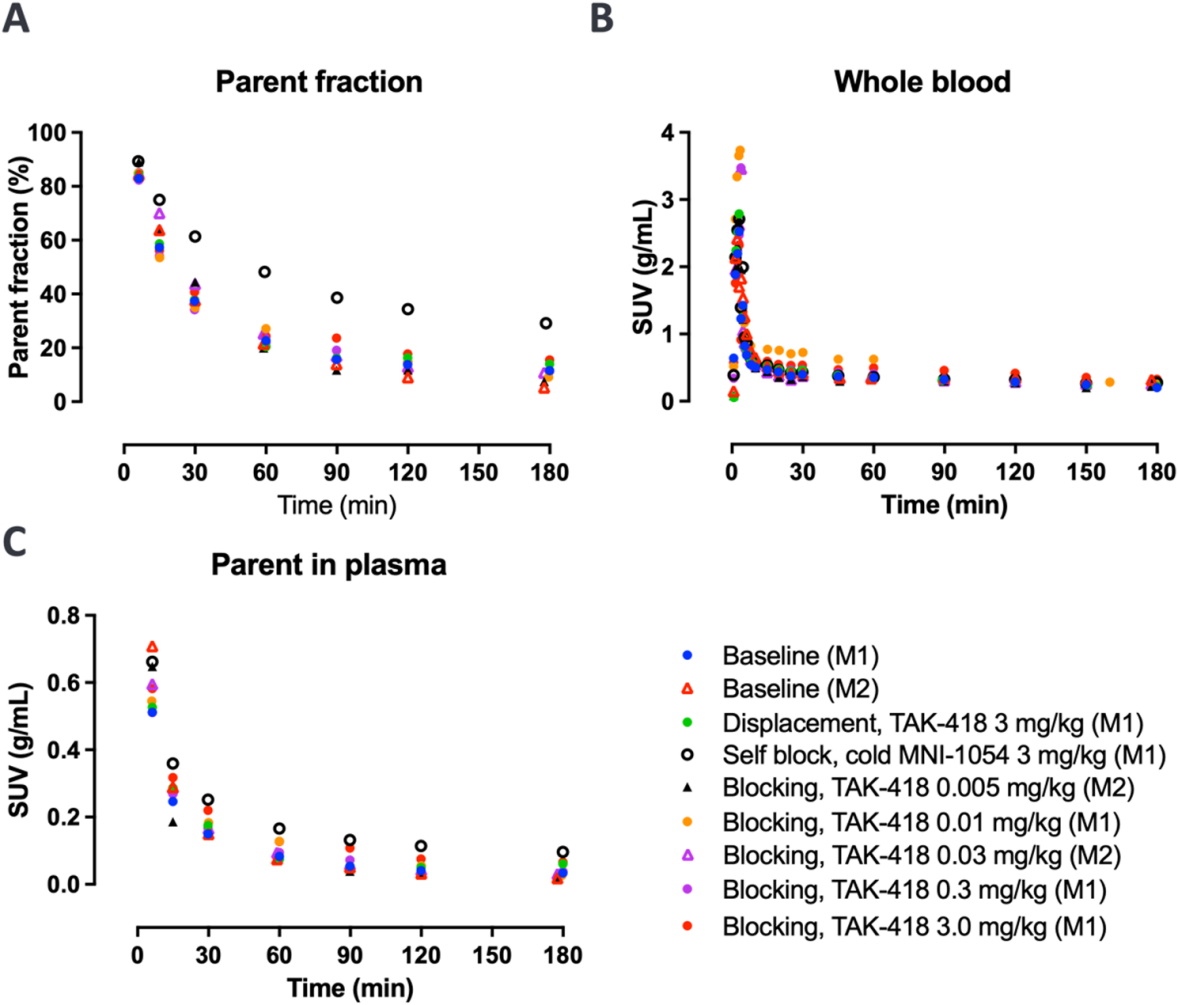
[^18^F]MNI1054% parent in arterial plasma (**A**), total activity concentration in arterial whole-blood (**B**), and [^18^F]MNI1054 activity in plasma (**C**) across brain studies in animal M1 and M2

[^18^F]MNI­1054 parent fraction in arterial plasma, as well as the whole blood and parent-in-plasma time courses are shown in Fig. 1 across brain imaging studies. Across baseline and blocking studies, parent fraction was ~42% at 30 min and ~14% at 180 min post-injection. The parent fraction and parent-in-plasma activity concentration levels were about 20% higher in the self-block study. Free fractions across studies ranged between 46 and 52%.

### Neuroanatomical distribution and binding kinetics of [^18^F]MNI­1054

**Fig. 2:**
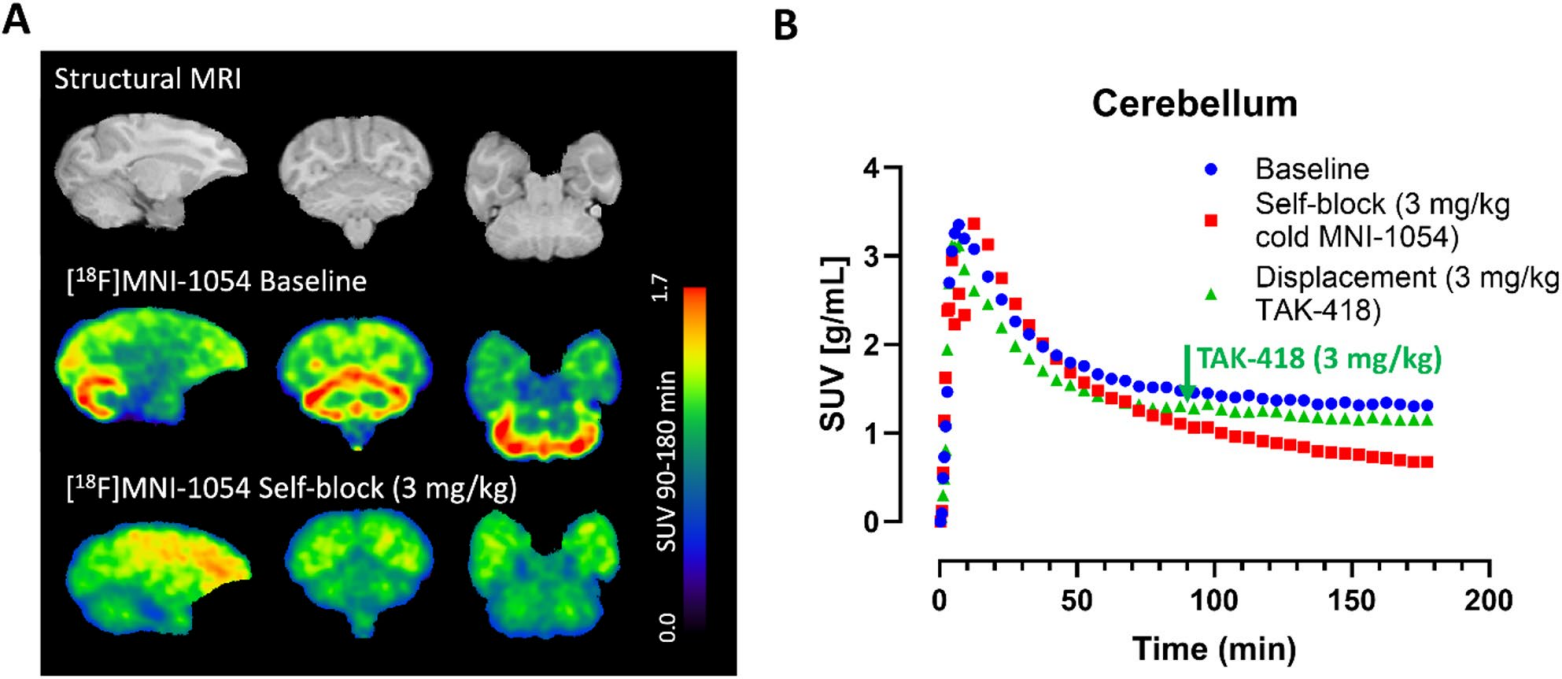
(**A**) Brain MRI (top panel) and summed SUV images of [^18^F]MNI-1054 (90–180 min post tracer injection) at baseline (middle) and after self-block with 3 mg/kg cold MNI-1054 (bottom panel) in M1. (**B**) Time-activity-curves (TACs) in cerebellum extracted from the baseline, self-block (3 mg/kg cold MNI-1054) and displacement (3 mg/kg TAK-418) studies in M1

[^18^F]MNI-1054 showed good brain penetrability with peak SUV values between 3 and 4 g/mL reached 7 to 12 min after administration in the baseline studies. The anatomical distribution of [^18^F]MNI-1054 was consistent with the known LSD1 regional distribution, with uptake highest in the cerebellum and lowest in the pons (Fig. 2A). Self-block with cold MNI­1054 (3.0 mg/kg) showed a reduction in specific binding of [^18^F]MNI­1054. The highest occupancy was observed in the cerebellum (96.1%), with other cortical and subcortical regions showing comparably high occupancy (78.1–92.8%) while occupancy in white matter was somewhat lower (approximately 69.3%) (Fig. 2B). Administration of 3.0 mg/kg TAK-418 90 min post tracer injection showed no observable change in the TACs, confirming the irreversible binding of [^18^F]MNI-1054 (Fig. 2B).

### Quantification of brain [^18^F]MNI-1054 PET

**Fig. 3:**
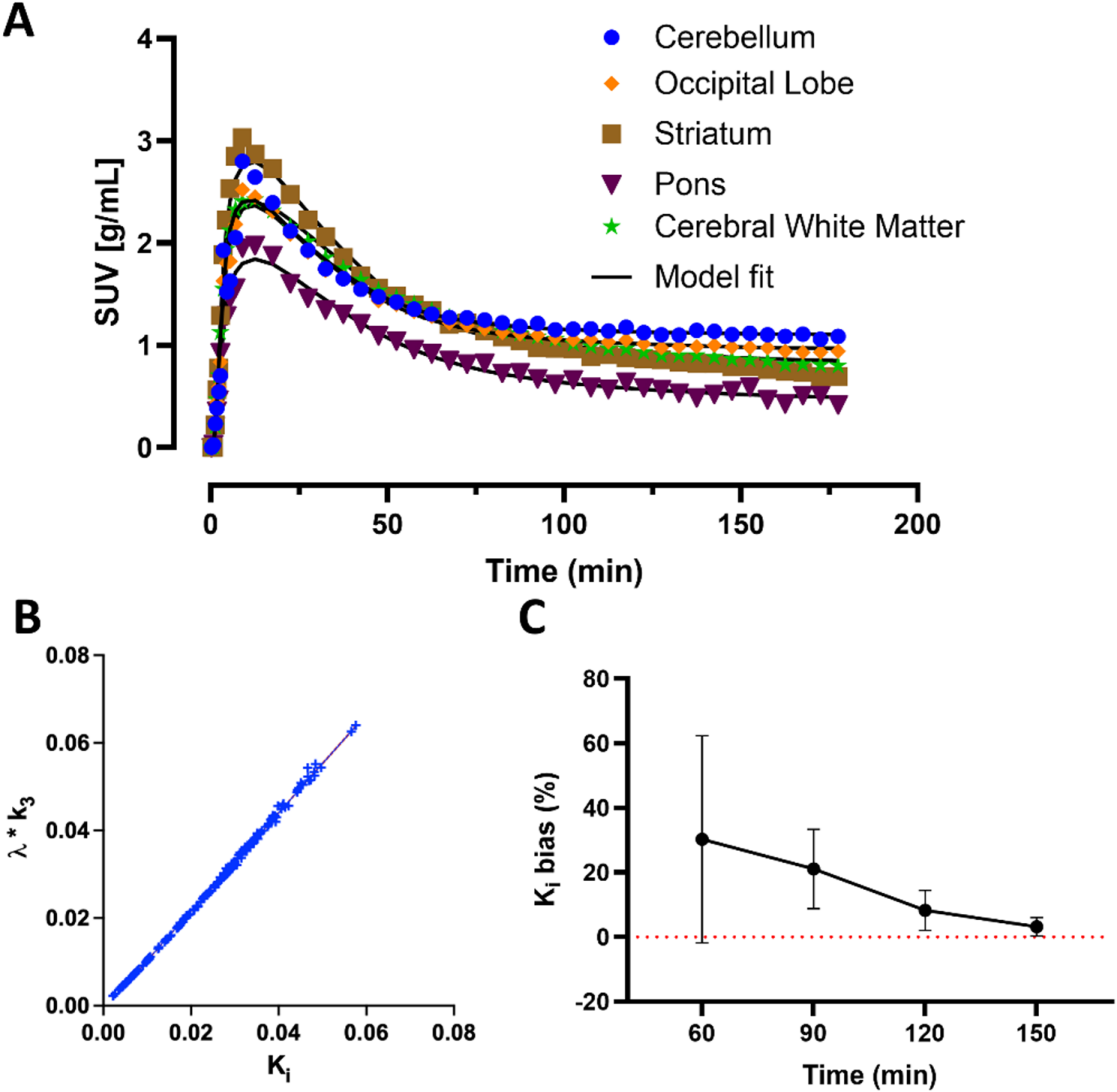
(**A**) Time activity curves from a baseline [^18^F]MNI-1054 scan (M2) for the Cerebellum, Frontal Lobe, Occipital Lobe, Striatum and Pons. Regional data are displayed alongside irreversible 2TCM fitting curves (solid lines). (**B**) Correlation between *K*_*i*_ and *λk*_3_ (*λ* = K_1_/k_2_), as estimated from irreversible 2TCM fitting, across all brain regions and studies. (**C**) Time stability of *K*_*i*_ estimates for the 2TCM for different scan durations in baseline studies. Error bars represent the standard deviation

The irreversible 2TCM fitted the [^18^F]MNI-1054 TACs well (Fig. 3A). Kinetic modeling confirmed that *K*_*i*_ was not flow-limited in the brain, with mean (SD) *k*_2_/*k*_3_ = 24.7 (28.6) (min: 6.1; max: 194.4) across all scans and regions, and agreed very well with *λ.k*_3_ (R^2^ = 0.999; Y = 1.123*X − 0.0009; *P* < 0.0001; Fig. 3B), confirming the choice of *K*_*i*_ as the primary quantitative outcome reflecting the density of LSD1 binding sites. *K*_*i*_ values estimated from Patlak analysis with t* = 30 min were linearly correlated with those obtained from compartmental modeling (*K*_*i, Patlak*_ = 0.9498* *K*_*i,2TCMirrev*_ – 0.004017; R^2^ = 0.927; *P* < 0.0001; see Fig. S.3), though *K*_*i, Patlak*_ values showed a small underestimation bias with respect to *K*_*i,2TCMirrev*_ (mean +/- SD difference: −0.00494 +/- 0.00348 mL/cm^3^/min). *K*_*i*_ values presented thereafter were obtained from the irreversible 2TCM. In the baseline scans, *K*_*i*_ was 0.047–0.058 mL/cm^3^/min in cerebellar gray matter, 0.023–0.045 mL/cm^3^/min in cortical and subcortical regions, 0.018–0.023 mL/cm^3^/min in the pons and 0.033–0.035 mL/cm^3^/min in cerebral white matter. Regional *K*_*i*_ did not substantially vary in the displacement study compared with baseline in the same animal. Figure 3**C** presents the time stability of *K*_*i*_ estimates across all analyzed regions for the irreversible 2TCM, showing a progressively increasing overestimation bias as scan time was reduced to 60 min. Average *K*_*i*_ bias was 8.3 +/- 6.1% (SD) for a 120-min scan duration, decreasing to 3.2 +/- 2.8% for a 150-min scan.

### Enzyme occupancy of TAK-418

**Fig. 4:**
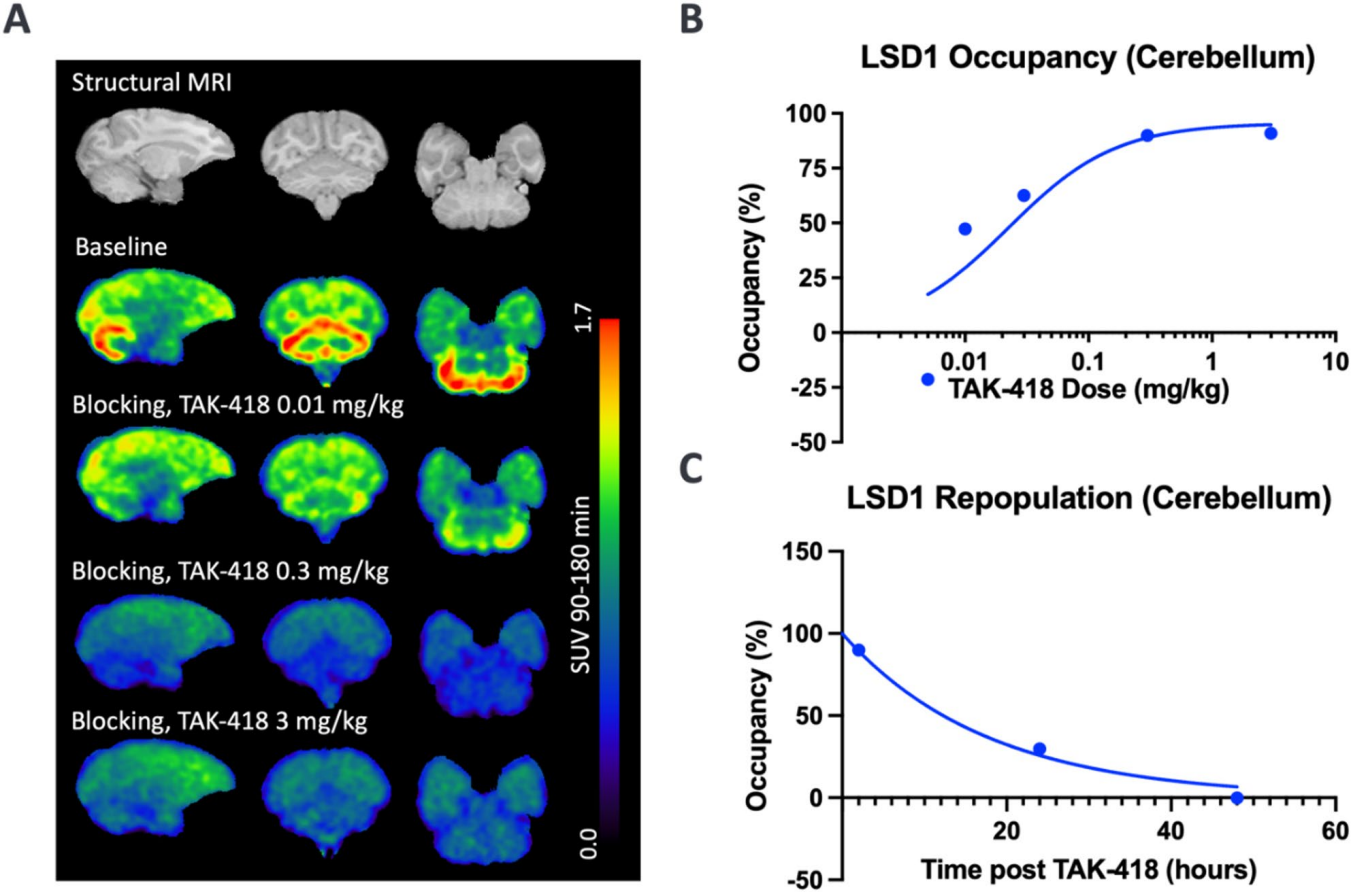
(**A**) Brain MRI (top panel) and summed SUV images (90–180 min post-injection) from M1 at baseline and following blockade with 0.01, 0.3 and 3 mg/kg TAK-418. (**B**) E_max_ model fit to enzyme occupancy values computed for the cerebellum. (**C**) Occupancy of TAK-418 in the cerebellum at 2- (M1), 24- (M2) and 48-hour (M2) scans along with single exponential fit, reflecting repopulation of unbound LSD1 enzyme

The blocking studies with TAK-418 resulted in a dose-dependent decrease in [^18^F]MNI-1054 specific binding, with a maximum occupancy of 90.9% observed at 3.0 mg/kg (Table 2). Maximal occupancy of TAK-418 at 3.0 mg/kg in other brain regions were lower (65.8–82.7%) but followed a similar dose-dependent reduction. An *E*_max_ model fit yielded estimates of *O*_max_ = 95.6% and *ED*_50_ = 0.0224 mg/kg (95% CIs [1e-4, 0.46] mg/kg) in cerebellum (Fig. 4B). Corresponding values for *O*_max_ and *ED*_50_ in other regions were 77.3% and 0.0279 mg/kg for the occipital cortex, and 73.3% and 0.046 mg/kg in the pons.

**Table 2:** [^18^F]MNI-1054 K_i_ (mL/cm^3^/min) and enzyme occupancy in the cerebellum following Blockade with TAK-418

TAK-418 Dose (mg/kg)	Animal #	K_i_ (mL/cm^3^/min)		Occupancy
		Baseline	2 h post-dose	
0.005	M2	0.047	0.057	−21.3%
0.01	M1	0.058	0.030	47.3%
0.03	M2	0.047	0.017	62.5%
0.3	M1	0.058	0.006	89.9%
3	M1	0.058	0.005	90.1%

### LSD1 enzyme repopulation

In the repopulation studies (M2), there were no detectable levels of TAK-418 in plasma at either 24- or 48-hours post-dose. LSD1 enzyme occupancy in the cerebellum was 29.8% at 24 h and − 0.05% at 48 h (Fig. 4C). This represents a significant repopulation of the LSD1 enzyme during this time frame, as a dose of 0.3 mg/kg TAK-418 occupied 89.9% of the available enzyme at 2 h post-TAK-418 dosing (M1). An exponential fit to these occupancy values yielded an estimated half-life for occupancy to decrease to zero, as a proxy for enzyme repopulation, of 12.28 h.

### [^18^F]MNI­1054 imaging in the testes

**Fig. 5:**
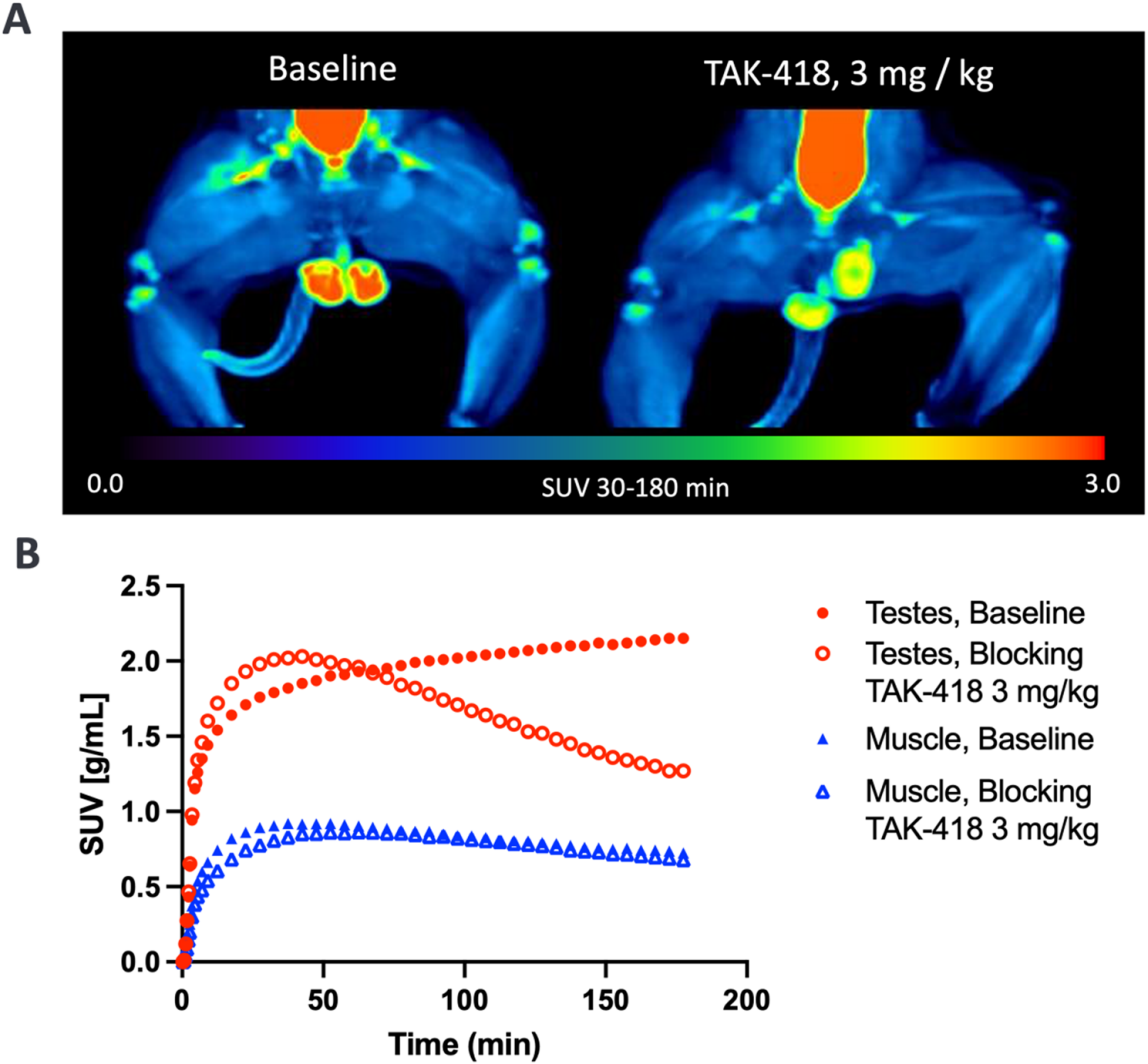
(**A**) Coronal [^18^F]MNI­1054 maximum intensity projection images of testes at baseline (left) and post blocking with 3.0 mg/kg TAK-418 (right). (**B**) Time activity curves in testes and muscle at baseline and post TAK-418 blocking

[^18^F]MNI-1054 binding in the testes, an organ with high LSD1 expression, was investigated with an additional baseline and blocking study in a male monkey (M3). Based on brain studies where full occupancy was observed, 3.0 mg/kg TAK-418 was chosen for blocking in the testes. Maximum intensity projection (MIP) images and TACs are shown in Fig. 5, demonstrating significant signal blockade in the testes compared with control leg muscle. However, point occupancy estimates could not be reliably estimated in the testes from *K*_*i*_ in that $\:{k}_{2}$ was approximately equal to $\:{k}_{3}$ at baseline, meaning that *K*_*i*_ reflected a mixture of blood flow and enzyme density. Interestingly, occupancy levels calculated from fractional changes in $\:{k}_{3}$ in the testes yielded an occupancy of 94.4%, which was similar to that observed in cerebellum at that dose for M1.

### Radiation dosimetry

Whole-body imaging revealed that [^18^F]MNI-1054 is eliminated via both hepatobiliary and urinary pathways. Examples of organ TACs are shown in Fig. S.4-S.6, along with whole body images (Fig. S.7). The [^18^F]MNI-1054 estimated absorbed doses in target organs in units of mSv/MBq are presented in Table 3 for the ICRP-89 adult male and female human phantom models. The gallbladder wall received the highest dose (0.177 mSv/MBq), followed by the urinary bladder (0.0916 mSv/MBq) using the no-bladder-void model. Based on the gallbladder wall as the dose-limiting organ, the maximal dose allowed for a single study per 21 CFR § 361.1 is 282.5 MBq. The estimated whole-body effective doses scaled from NHP to human were 0.0272 mSv/MBq for females and 0.0267 mSv/MBq for males, which is comparable to other ^18^F-labeled small molecule tracers. The female value is equivalent to 5.03 mSv per 185 MBq of [^18^F]MNI-1054 injected.

**Table 3:** [^18^F]MNI­1054 target organ and radiation exposure summary for ICRP­89 adult male and female models, and whole-body effective dose (ED) with ICRP­103 tissue weighting factors

Target organ	Dose (mSv/MBq)		
	Adult male	Adult female	Mean
Adrenals	1.87E-02	2.24E-02	2.06E-02
Brain	9.69E-03	1.22E-02	1.09E-02
Breasts	ΝΑ	1.06E-02	1.06E-02
Esophagus	1.49E-02	1.75E-02	1.62E-02
Eyes	7.78E-03	9.29E-03	8.54E-03
Gallbladder wall	6.57E-02	2.88E-01	1.77E-01
Left colon	1.62E-02	3.02E-02	2.32E-02
Small intestine	2.08E-02	6.01E-02	4.05E-02
Stomach wall	1.42E-02	1.69E-02	1.56E-02
Right colon	2.37E-02	6.29E-02	4.33E-02
Rectum	1.60E-02	1.88E-02	1.74E-02
Heart wall	4.90E-02	4.98E-02	4.94E-02
Kidneys	4.63E-02	5.28E-02	4.96E-02
Liver	3.02E-02	2.29E-02	2.66E-02
Lungs	4.01E-02	6.16E-02	5.09E-02
Ovaries	ΝΑ	1.66E-02	1.66E-02
Pancreas	1.48E-02	2.12E-02	1.80E-02
Prostate	1.78E-02	ΝΑ	1.78E-02
Salivary glands	9.10E-03	1.01E-02	9.60E-03
Red marrow	1.65E-02	2.01E-02	1.83E-02
Osteogenic cells	1.21E-02	1.36E-02	1.29E-02
Spleen	1.84E-02	2.77E-02	2.31E-02
Testes	3.17E-02	ΝΑ	3.17E-02
Thymus	1.44E-02	1.88E-02	1.66E-02
Thyroid	1.14E-02	1.32E-02	1.23E-02
Urinary bladder wall	1.24E-01	5.92E-02	9.16E-02
Uterus	ΝΑ	1.92E-02	1.92E-02
*Total body*	*1.16E-02*	*1.53E-02*	*1.35E-02*
*ED (ICRP-103)*	*2.67E-02*	*2.72E-02*	*2.70E-02*

## Discussion

[^18^F]MNI-1054 readily entered the rhesus monkey brain and its distribution was highest in the cerebellum, intermediate in cortical regions (e.g., occipital, frontal), lower in subcortical regions and lowest in the pons which represents an anatomical binding profile that correlates well with known LSD1 expression levels [14]. An irreversible 2TC model was successfully used to estimate *K*_*i*_ as the primary outcome measure in the brain, reflecting the density of LSD1 binding sites. [^18^F]MNI-1054 did not displace after TAK-418 administration, consistent with irreversible tracer kinetics.

Non-radiolabeled MNI-1054 (3 mg/kg) blocked most of the specific signal, and while modest regional differences in TAK-418 occupancy were observed, the trends across regions were consistent. The novel LSD1 inhibitor TAK-418 produced a dose-dependent reduction in brain uptake of [^18^F]MNI-1054 and in estimated *K*_*i*_ values, with an *O*_max_ of 95.6% being observed in the cerebellum, which was the selected target region based on having the highest level of specific binding. The *ED*_50_ of TAK-418 was estimated at ~0.0224 mg/kg in the cerebellum and ~0.0279 mg/kg in the occipital cortex.

The lowest administered dose (0.005 mg/kg) resulted in an unexpectedly large negative occupancy value (Table 2). A closer examination of the modeling results indicates that this effect was driven in part by a higher *K*_*1*_*/k*_*2*_ ratio in the blocking scan rather than by an increased enzyme binding reflected through an increase in k_3_. Interestingly, occupancy calculations based on fractional changes in *k*_*3*_ produced a more plausible estimate at Occ(%) = 2.4% for 0.005 mg/kg. This underscores the potential limitation in using *K*_*i*_ for tracer quantification since it can be influenced not only by binding changes but also by variations in blood flow and permeability.

Due to the irreversible binding of both [^18^F]MNI-1054 and TAK-418, as well as the rapid washout of TAK-418, reduced levels of enzyme occupancy derived from scans at 24- and 48-hours post-TAK-418 dosing could be used to estimate the rate of enzyme repopulation in the brain. This corresponds to the production of a new pool of LSD1 enzyme unbound to TAK-418, in parallel to the degradation of the bound and unbound pools that were present immediately following TAK-418 administration. For these studies, TAK-418 was administered as a 5-minute intravenous bolus, reaching a peak plasma concentration at the end of this bolus period at all doses, and decaying rapidly thereafter. At the 0.3 mg/kg dose used for repopulation studies, the plasma concentration was 0.45% of its peak value after 5 h and below the level of detection after 24 and 48 h. We thus assumed that there was no further contribution of TAK-418 binding at those last two time points and used a simple exponential model to estimate the decay in enzyme occupancy, i.e., the proportion of TAK-418-bound LSD1, as a proxy for the enzyme repopulation rate. Although the 2-hour data point was obtained in a different animal, the exponential model fit the three data points well and yielded an estimate of the LSD1 enzyme repopulation rate in the rhesus macaque of 12.28 h.

The ability of [^18^F]MNI-1054 to gauge peripheral tissue occupancy in addition to central occupancy was also assessed, with the testes chosen as the tissue of interest due to its high endogenous expression of LSD1. A baseline scan showed that [^18^F]MNI-1054 demonstrated high testes uptake (SUV ~2), with pretreatment of TAK-418 (3 mg/kg) causing a substantial reduction. No such changes were observed in muscle which was chosen as a surrogate reference region to ensure that there was no significant change in either flow, metabolism or clearance which could affect tissue uptake. This illustrates that [^18^F]MNI-1054 has the potential to characterize peripheral point occupancies, though additional work will be needed to fully evaluate this possibility including selecting the most appropriate binding parameters.

The radiation dosimetry profile of [^18^F]MNI-1054 was found to be comparable to other ^18^F-labeled radiopharmaceuticals. [^18^F]MNI­1054 elimination takes place via both urinary (primary) and hepatobiliary pathways. The estimated whole-body effective doses (ED, ICRP-103), scaled from NHP to human, were 0.0272 mSv/MBq (adult female), and 0.0267 mSv/MBq (adult male). These whole-body dosimetry values indicate that up to nine [^18^F]MNI-1054 injections of 185 MBq each could be performed per year. However, the gallbladder wall (i.e., limiting organ) dose, which can be physiologically mitigated, limits the number of injections to four per year according to 21 CFR § 361.1.

An important consideration inherent to preclinical PET studies is the use of anesthesia, particularly for long dynamic acquisitions such as the 180-minute scans employed here. Anesthetics such as isoflurane can indeed impact cerebral physiology such as by altering cerebral blood flow and metabolism [27–29]. While we cannot exclude an absolute effect of anesthesia on imaging findings compared to an awake state, all vital signs (e.g., heart rate, blood pressure, ETC02, temperature) remained stable across the full scan duration in all experiments, showing no evidence of a changing physiological state. Also, the steady-state kinetic models used in this study fitted the PET data very well across the full 3-hour of acquisition, further supporting physiological stability during PET imaging. Lastly, physiological signs remained largely consistent across baseline and blocking sessions in the same animal, minimizing the likelihood that variability in anesthesia between sessions confounded occupancy estimates. Therefore, while anesthesia might have altered absolute physiological parameters compared to an awake state, the data provide evidence for stable systemic physiology *within* and *between* scans, supporting the validity of reported findings.

## Conclusion

[^18^F]MNI-1054 showed acceptable brain penetrance, kinetics and radiation dosimetry comparable to other ^18^F-labelled small molecule PET tracers. The specific binding was demonstrated to be irreversible and modeled using an irreversible two-tissue compartment model, yielding *K*_*i*_ as the binding parameter. The highest specific signal was found in cerebellar gray matter, which represents a suitable target region. The specific brain signal was blocked in a dose-dependent fashion by the novel, and molecularly distinct, LSD1 inhibitor TAK-418, with estimated *O*_max_ = 95.6% and *ED*_50_ = 0.0224 mg/kg in cerebellum. Scans at later time points enabled an estimate of the LSD1 enzyme repopulation half-life of 12.28 h in the rhesus macaque. Overall, these findings demonstrate the suitability of [^18^F]MNI-1054 as a viable PET probe to assess the binding profile of novel LSD1 inhibitors in the brain, and support further evaluation in humans.

## Supplementary Information

Supplementary Material 1.

## Data Availability

The data that support the findings of this study are available from the corresponding author upon reasonable request.

## References

[1] Klibaner-Schiff E, et al. Environmental exposures influence multigenerational epigenetic transmission. Clin Epigenetics. 2024;16(1):145. 10.1186/s13148-024-01762-3. 39420431 10.1186/s13148-024-01762-3 PMC11487774

[2] Neelamegam R, et al. Brain-Penetrant LSD1 inhibitors can block memory consolidation. ACS Chem Neurosci. 2012;3(2):120–8. 10.1021/cn200104y. 22754608 10.1021/cn200104y PMC3382965

[3] Vallianatos CN, Iwase S. Disrupted intricacy of histone H3K4 methylation in neurodevelopmental disorders. Epigenomics. 2015;7(3):503–19. 10.2217/epi.15.1. 26077434 10.2217/epi.15.1 PMC4501478

[4] The Network and Pathway Analysis Subgroup of the Psychiatric Genomics Consortium. Psychiatric genome-wide association study analyses implicate neuronal, immune and histone pathways, Nat. Neurosci*.* Feb. 2015;18(2);199–209. 10.1038/nn.3922 10.1038/nn.3922 PMC4378867 25599223

[5] Huang H-S, et al. Prefrontal dysfunction in schizophrenia involves mixed-lineage leukemia 1-regulated histone methylation at GABAergic gene promoters. J Neurosci. 2007;27(42):11254–62. 10.1523/JNEUROSCI.3272-07.2007. 17942719 10.1523/JNEUROSCI.3272-07.2007 PMC6673022

[6] Vashishtha M et al. Targeting H3K4 trimethylation in Huntington disease. Proc. Natl. Acad. Sci*.* 2013:110(32). 10.1073/pnas.1311323110 10.1073/pnas.1311323110 PMC3740882 23872847

[7] Wang J, et al. LSD1n is an H4K20 demethylase regulating memory formation via transcriptional elongation control. Nat Neurosci. 2015;18(9):1256–64. 10.1038/nn.4069. 26214369 10.1038/nn.4069 PMC4625987

[8] Bjornsson HT, et al. Histone deacetylase Inhibition rescues structural and functional brain deficits in a mouse model of Kabuki syndrome. Sci Transl Med. 2014. 10.1126/scitranslmed.3009278. 25273096 10.1126/scitranslmed.3009278 PMC4406328

[9] Zhang L, et al. Inhibition of KDM1A activity restores adult neurogenesis and improves hippocampal memory in a mouse model of Kabuki syndrome. Molecular Therapy - Methods & Clinical Development. 2021;20:779–91. 10.1016/j.omtm.2021.02.011. 33738331 10.1016/j.omtm.2021.02.011 PMC7940709

[10] Baba R, et al. LSD1 enzyme inhibitor TAK-418 unlocks aberrant epigenetic machinery and improves autism symptoms in neurodevelopmental disorder models. Sci Adv. 2021;7(11):eaba1187. 10.1126/sciadv.aba1187. 33712455 10.1126/sciadv.aba1187 PMC7954450

[11] Christopher MA, et al. LSD1 protects against hippocampal and cortical neurodegeneration. Nat Commun. 2017;8(1):805. 10.1038/s41467-017-00922-9. 28993646 10.1038/s41467-017-00922-9 PMC5634471

[12] Engstrom AK, et al. The inhibition of LSD1 via sequestration contributes to tau-mediated neurodegeneration. Proc Natl Acad Sci U S A. 2020;117(46):29133–43. 10.1073/pnas.2013552117. 33139560 10.1073/pnas.2013552117 PMC7682552

[13] Song Y, Yu B. Leveraging non-enzymatic functions of LSD1 for novel therapeutics. Trends Pharmacol Sci. 2025;46(3):204–19. 10.1016/j.tips.2025.01.006. 39966067 10.1016/j.tips.2025.01.006

[14] Hattori Y, et al. Design, synthesis, and evaluation of (2-aminocyclopropyl)phenyl derivatives as novel positron emission tomography imaging agents for lysine-specific demethylase 1 in the brain. J Med Chem. 2021;64(7):3780–93. 10.1021/acs.jmedchem.0c01937. 33729758 10.1021/acs.jmedchem.0c01937

[15] Matsuda S, et al. Design, synthesis, and evaluation of [^18^F]T-914 as a novel positron-emission tomography tracer for lysine-specific demethylase 1. J Med Chem. 2021;64(17):12680–90. 10.1021/acs.jmedchem.1c00653. 34423983 10.1021/acs.jmedchem.1c00653

[16] Baba R, et al. Investigating the therapeutic potential of LSD1 enzyme activity-specific inhibition by TAK-418 for social and memory deficits in rodent disease models. ACS Chem Neurosci. 2022;13(3):313–21. 10.1021/acschemneuro.1c00713. 35061371 10.1021/acschemneuro.1c00713

[17] Lehnert W, Meikle SR, Siegel S, Bailey D, Banati R, Rosenfeld AB. Evaluation of Transmission Methodology for the microPET Focus 220 Animal Scanner, in IEEE Nuclear Science Symposium Conference Record, 2005, Wyndham El Conquistador Resort, Puerto Rico: IEEE. 2005, pp. 2519–2523. 10.1109/NSSMIC.2005.1596852

[18] Jenkinson M, Beckmann CF, Behrens TEJ, Woolrich MW, Smith SM. FSL. Neuroimage. 2012;62(2):782–90. 10.1016/j.neuroimage.2011.09.015. 21979382 10.1016/j.neuroimage.2011.09.015

[19] Cox RW. AFNI: Software for analysis and visualization of functional magnetic resonance neuroimages. Comput Biomed Res. 1996;29(3):162–73. 10.1006/cbmr.1996.0014. 8812068 10.1006/cbmr.1996.0014

[20] Rohlfing T, et al. The INIA19 template and neuromaps atlas for primate brain image parcellation and Spatial normalization. Front Neuroinformatics. 2012;6. 10.3389/fninf.2012.00027. 10.3389/fninf.2012.00027 PMC3515865 23230398

[21] Jung B, et al. A comprehensive macaque fMRI pipeline and hierarchical atlas. Neuroimage. 2021;235:117997. 10.1016/j.neuroimage.2021.117997. 33789138 10.1016/j.neuroimage.2021.117997 PMC9272767

[22] Seidlitz J, et al. A population MRI brain template and analysis tools for the macaque. Neuroimage. 2018;170:121–31. 10.1016/j.neuroimage.2017.04.063. 28461058 10.1016/j.neuroimage.2017.04.063 PMC5660669

[23] Moré JJ. The Levenberg-Marquardt algorithm: implementation and theory. In: Watson GA, editor. Numerical analysis. Lecture Notes in Mathematics. Volume 630. vol. 630., Berlin, Heidelberg: Springer Berlin Heidelberg; 1978. pp. 105–16. 10.1007/BFb0067700.

[24] Patlak CS, Blasberg RG, Fenstermacher JD. Graphical evaluation of blood-to-brain transfer constants from multiple-time uptake data. J Cereb Blood Flow Metab. 1983;3(1):1–7. 10.1038/jcbfm.1983.1. 6822610 10.1038/jcbfm.1983.1

[25] Nozaki S, et al. Selective lysine-specific demethylase 1 inhibitor, NCL1, could cause testicular toxicity via the regulation of apoptosis. Andrology. 2020;8(6):1895–906. 10.1111/andr.12846. 32598553 10.1111/andr.12846 PMC7689788

[26] Preface E, Summary, Glossary. Ann ICRP. Apr. 2007;37(2–4):9–34. 10.1016/j.icrp.2007.10.003.

[27] Reinstrup P, Ryding E, Algotsson L, Messeter K, Asgeirsson B, Uski T. Distribution of cerebral blood flow during anesthesia with isoflurane or halothane in humans. Anesthesiology. 1995;82(2):359–66. 10.1097/00000542-199502000-00006. 7856894 10.1097/00000542-199502000-00006

[28] Toyama H, et al. Evaluation of anesthesia effects on [18F]FDG uptake in mouse brain and heart using small animal PET. Nucl Med Biol. 2004;31(2):251–6. 10.1016/S0969-8051(03)00124-0. 15013491 10.1016/S0969-8051(03)00124-0

[29] Hildebrandt IJ, Su H, Weber WA. Anesthesia and other considerations for in vivo imaging of small animals. ILAR J. 2008;49(1):17–26. 10.1093/ilar.49.1.17. 18172330 10.1093/ilar.49.1.17

