# Supplementary material for: Evaluation of [18F]MNI-1054, a novel PET ligand for lysine-specific histone demethylase 1A (LSD1), in non-human primates
Source: EJNMMI Res. 2025 Dec 6;16:8. doi: 10.1186/s13550-025-01350-3 (PMC12796071; doi:10.1186/s13550-025-01350-3)
Supplement: Supplementary file 1 — Supplementary Material 1. [file 13550_2025_1350_MOESM1_ESM.docx]

**SUPPLEMENTARY MATERIAL**

**Evaluation of [^18^F]MNI-1054, a novel PET ligand for lysine-specific histone demethylase 1A (LSD1), in non-human primates**

Yoann Petibon^1^, Akihiro Takano^2,**^, Adam J Schwarz^1,**^, Ozlem Yardibi^1^, Christine Sandiego^3^, Olivier Barret^3^, Cristian Constantinescu^3^, Johannes Tauscher^1^, Paul McQuade^1,*^

*^1^Takeda Development Center Americas, Inc., Cambridge MA, USA*

*^2^Takeda Pharmaceutical Company Ltd., Osaka, Japan*

*^3^Invicro LLC, New Haven CT, USA*

***At time of study*

***Corresponding author:**

Paul McQuade, Takeda Development Center Americas, Inc.,125 Binney St., Cambridge, MA 02139, USA.

Email: [paul.mcquade@takeda.com](mailto:paul.mcquade@takeda.com); Phone: (617)444-1453

This study was funded by Takeda Pharmaceutical Company Ltd.

1. **[^18^F]MNI-­1054 Synthesis**

[^18^F]MNI-­1054 was prepared as shown in **Figure S.1.** using a commercial synthesizer, GE TRACERlab™ FX­FN [1].

Figure S.1: Radiosynthesis of [^18^F]MNI­-1054.

In a typical procedure, [^18^F]fluoride in a shipping vial (target water obtained from a commercial cyclotron facility) is transferred onto and trapped on an ion exchange cartridge. It is then eluted with a solution of potassium carbonate and Kryptofix® 222 into the reaction vessel (RV1) of the TRACERlab™ module. The solution is first evaporated by heating at 95 °C for 4 min under vacuum and helium flow. Acetonitrile (1 mL) is added to RV1 and the evaporation is continued under the same conditions for 2 min under vacuum and helium flow. After a second addition of acetonitrile (1 mL), the final evaporation is carried out at 95 °C for 2 min under vacuum and helium flow. The reactor is then cooled to 60 °C. A solution of the precursor (1 mg, MNI-1053) in anhydrous dimethylsulfoxide is added to the reaction vessel and the reaction mixture is heated at 150°C for 10 min. The reactor is cooled to 90 °C and hydrochloric acid (1M) is added and heated at 90 °C for 4 min. The reactor is cooled to 40 °C, and the contents are transferred into the loop-loading vial (RV1), preloaded with sodium hydroxide (1M). The reactor is rinsed with HPLC mobile phase and the rinse is transferred into RV2. The contents of RV2 are transferred into the HPLC injector loop for purification.

Purification is performed by HPLC using a semi-preparative C18 column (Waters XBridge C18 column (5 µm, 250 x 10 mm)) and eluted with a mixture of acetonitrile/ammonium acetate solution (20 mM) (25/75, v/v) at a flow rate of 4 mL/min. The product fraction is collected in Flask1, containing 20 mL of ascorbic acid in WFI. The diluted product mixture is passed through a C18 solid-phase extraction cartridge and the cartridge is rinsed with 10 mL of ascorbic acid in WFI. The radiolabeled product is eluted from the SPE cartridge with 1 mL of 200-proof USP grade ethanol into the formulation flask, pre-loaded with 10 mL of formulation base. The cartridge is rinsed with 4 mL of formulation base and the rinse is mixed with the contents of the formulation flask. The resulting solution is passed through a sterilizing 0.2­μm membrane filter into a sterile, filter-vented vial (final product vial, FPV), pre-filled with 15 mL of normal saline.

Quality control testing includes visual inspection of appearance. Identity, chemical, and radiochemical purity are determined by HPLC; strength is measured by gamma assay; filter integrity, pyrogen content, and sterility are determined by compendial tests per USP; residual solvents and ethanol content are determined by GC; residual Kryptofix® 222 is determined by TLC; pH is measured using pH paper. All tests, except inoculation of product in two media for sterility, are performed before the product is released. Inoculation is performed within 30 h after end of synthesis.

[^18^F]MNI-1054 was produced with a radiochemical purity of 99.8 ± 0.3% and a molar activity of 3585 ± 1952 Ci/mmol (n=14). For the imaging studies, the average mass of MNI-1054 administered was 0.6 ± 0.3 µg.

1. **Arterial blood sampling and blood data analysis**

*Arterial Blood Sampling and Radioactivity Counting*

Arterial blood samples (1 mL or **3.5 mL**) were drawn at 0.75, 1.5, 2.25, 3, 3.75, 4, 5, **6**, 8, 10, **15**, 20, 25, **30**, 45, **60, 90, 120**, 150, and **180**min post-injection to measure parent fraction, total plasma and whole blood activity. The larger samples were drawn for analysis of parent fraction. Aliquots of 200 µL of whole blood were counted in a gamma counter (Wallac 2480 Wizard^2^® Automatic Gamma Counter, Perkin-Elmer, Waltham, Massachusetts) for 1 min (400-1400 keV window). All blood samples were centrifuged for 10 min at 3,000 *g* and the plasma separated. Aliquots of 200 µL of plasma were counted in the gamma counter for 1 min (400-1400 keV window).

*Arterial Plasma Radiometabolite Analysis*

The fraction of unchanged radiotracer in plasma was determined by HPLC at selected time points. The metabolite analysis was performed using an HPLC system consisting of a Phenomenex Luna C18(2) (10 x 250 mm) column eluted with a mobile phase consisting of a mixture of acetonitrile/water/triethylamine (60/40, 0.2%) at a flow rate of 4 mL/min. Plasma samples were processed by acetonitrile denaturation, treating 1 mL of plasma with 1 mL of acetonitrile. After vigorous mixing and centrifugation at 3,000 *g* for 10 min, the supernatant was transferred to an autosampler vial and injected onto HPLC. Sample vials and pellets were counted in the gamma counter for 1 min to calculate the extraction percentage. Gamma chromatograms were analyzed by integration of all radioactive peaks. The percentage of parent compound was calculated by dividing the area under the peak representing parent compound by the sum of the area of all radioactive peaks.

*Plasma Protein Binding (Free Fraction)*

For the determination of plasma free fraction (the fraction of radioligand that is not protein bound, f_P_), 200­µL aliquots of plasma spiked with the radiotracer (~2 µCi for 1 mL of plasma) were pipetted in duplicates into ultrafiltration units (Amicon Centrifree 30, Millipore) and centrifuged at 20°°C for 20 min at 3,000 g. The radioactivities of the ultrafiltrate (50­µL aliquot) and the filtration unit were counted. The procedure was repeated in duplicate. The plasma free fraction was calculated as the ratio of the ultrafiltrate activity concentration to the plasma activity concentration and expressed as the average of two measurements.

1. **Brain volume of interest (VOI) masks**

**
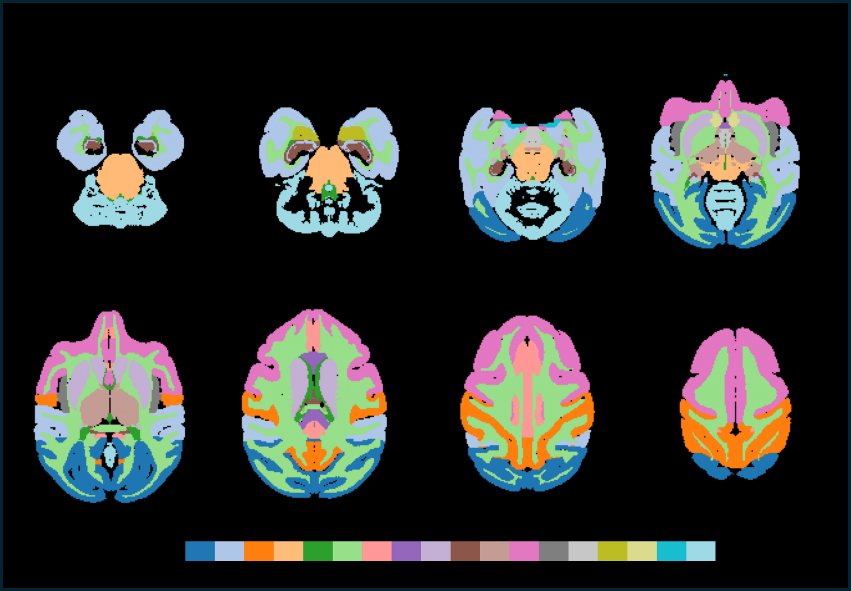
**

**Figure S.2. Visualization of the brain volume of interest (VOI) masks used for quantitative analysis. The presented VOIs were derived based on the anatomical parcellations of the INIA19 template (Rolfing et al Front. Neuroinformatics, vol. 6, 2012, doi: 10.3389/fninf.2012.00027). Masks are shown in the INIA19 template space.**

1. **Irreversible 2TC modeling vs. Patlak graphical analysis**

**Figure S.3.: Correlation plot between K_i_ values estimated from compartmental modeling (irreversible 2TCM) and those estimated from Patlak analysis, pooling data across all studies and brain regions.**

1. **Radiation dosimetry**

Figure S.4. [^18^F]MNI­1054 organ time activity curves expressed in [%Injected activity] acquired from a male rhesus macaque.

Figure S.5. [^18^F]MNI­1054 organ time activity curves expressed in [%Injected activity] acquired from a female rhesus macaque.

Figure S.6. Decay corrected uptake of [^18^F]MNI­1054 in source organs for both male and female rhesus macaque.


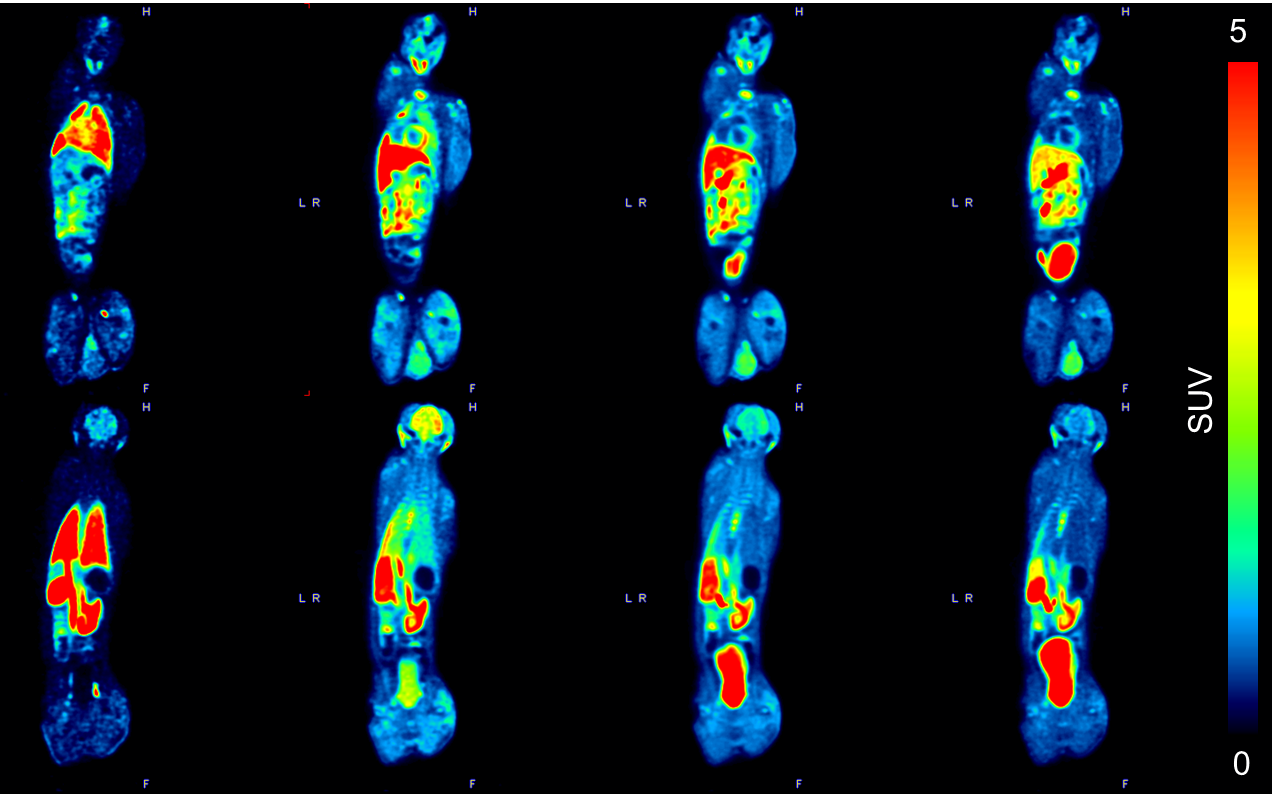


**Figure S.7. PET images (scaled to SUV) in 2 different coronal sections (4.28 cm apart) through whole body of male rhesus at ~ 3 min, 26 min, 99 min, and 228 min from tracer injection (left to right). Top images highlight heart, lungs, gallbladder, intestines, urinary bladder and testes. Bottom images highlight brain, lungs, kidneys, and urinary bladder**

**References**

[1] S. Matsuda *et al.*, “Design, Synthesis, and Evaluation of [^18^ F]T-914 as a Novel Positron-Emission Tomography Tracer for Lysine-Specific Demethylase 1,” *J. Med. Chem.*, vol. 64, no. 17, pp. 12680–12690, Sept. 2021, doi: 10.1021/acs.jmedchem.1c00653.
